# Lysosomal Vesicles, Giant Granules, and Erythrophagocytosis in Chédiak-Higashi Syndrome

**DOI:** 10.4274/tjh.2013.0210

**Published:** 2014-06-10

**Authors:** Burçin Beken, Şule Ünal, Fatma Gümrük

**Affiliations:** 1 Hacettepe University Faculty of Medicine, Department of Pediatrics, Ankara, Turkey

**Keywords:** Chediak-Higashi syndrome, Giant granules, Erytrophagocytosis

## 1. QUIZ IN HEMATOLOGY

A 3-year-old boy presented with recurrent infections. Physical examination revealed hepatosplenomegaly, bilateral cervical lymphadenopathy, silvery gray hair, and bilateral nystagmus. Giant granules in lymphocytes, monocytes, and granulocytes were seen on blood smear (Figure A). Bone marrow aspirate exhibited erythrophagocytosis and numerous giant granules of predominantly myeloid lineage (Figure B). Examination of the hair showed an irregular distribution of large and small pigment clumps (Figure C). 

## 

Chédiak-Higashi syndrome (CHS) is a rare autosomal recessive disorder characterized by partial oculocutaneous albinism, recurrent infections, and mild bleeding tendency [1]. Mutations in the lysosomal trafficking regulator gene (LYST) localized to chromosome 1q42-q44 are responsible for the disease [2]. Lysosomes of hematopoietic cells, particularly granulocytes and monocytes, are enlarged to form vesicles [3]. Giant inclusions in hematopoietic cells are the most reliable diagnostic clinical criterion for CHS. The main differential diagnosis is Griscelli syndrome, a rare autosomal recessive disorder caused by mutations in the MYO5A or RAB27A genes. This syndrome also manifests with partial albinism and immunodeficiency and it progresses towards the accelerated phase as in CHS, but it differs from CHS in view of the absence of giant intracytoplasmic granules in the leukocytes or by genetic analysis [4,5]. Approximately 85% of CHS patients develop a lymphoproliferative infiltration called “accelerated phase” compatible with hemophagocytic lymphohistiocytosis [1]. 

## Figures and Tables

**Figure A:**
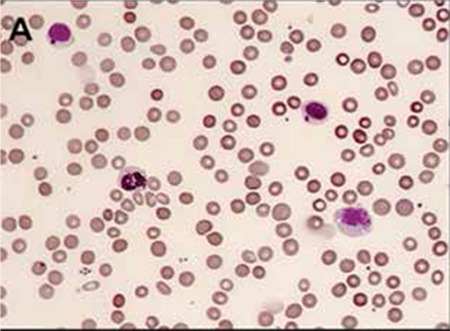
Blood smear exhibiting giant granules in granulocytes and lymphocytes.

**Figure B:**
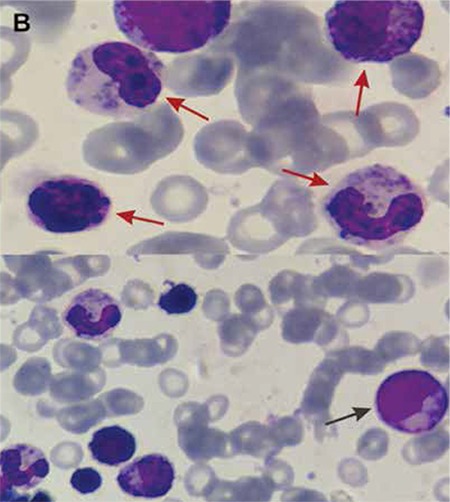
Bone marrow aspirate smears showing giant lysosomal granules within myeloid precursors (red arrows) and hemophagocytosis (black arrow).

**Figure C:**
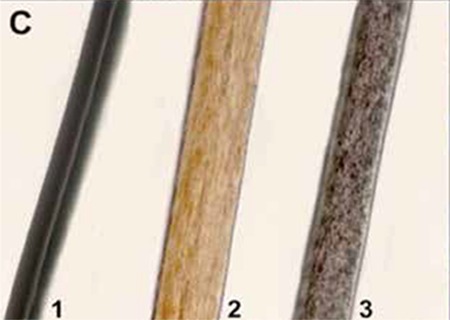
1) Normal dark hair, 2) normal blonde hair, 3) patient’s hair.
